# Supplementary material for: Primary care utilization among telehealth users and non-users at a large urban public healthcare system
Source: PLoS One. 2022 Aug 5;17(8):e0272605. doi: 10.1371/journal.pone.0272605 (PMC9355262; doi:10.1371/journal.pone.0272605)
Supplement: S1 Table — (DOCX) [file pone.0272605.s001.docx]

**Supplemental information**

**S1 Table. Full regression model for number of primary care visits by telehealth user status**

|  | Incidence rate ratio [95% confidence interval] | Standard error | p |
| --- | --- | --- | --- |
| Telehealth user status |  |  |  |
| Telehealth Non-user | Reference | Reference | Reference |
| Telehealth user | 1.44 [1.43, 1.45] | 0.0042 | <0.001 |
| Age, years |  |  |  |
| 18-44 | Reference | Reference | Reference |
| 45-64 | 1.11 [1.10, 1.12] | 0.0039 | <0.001 |
| ≥65 | 1.14 [1.13, 1.15] | 0.0054 | <0.001 |
| Sex |  |  |  |
| Male | Reference | Reference | Reference |
| Female | 1.03 [1.03, 1.04] | 0.0028 | <0.001 |
| Race/Ethnicity |  |  |  |
| White | Reference | Reference | Reference |
| Black | 1.02 [1.01, 1.03] | 0.0053 | 0.001 |
| Hispanic | 0.97 [0.95, 0.98] | 0.0078 | <0.001 |
| Asian/Pacific Islander | 1.02 [1.01, 1.04] | 0.0069 | 0.001 |
| Something Else | 1.00 [0.99, 1.01] | 0.0058 | 0.61 |
| Language |  |  |  |
| English | Reference | Reference | Reference |
| Spanish | 1.05 [1.04, 1.07] | 0.0080 | <0.001 |
| Something Else | 1.02 [1.01, 1.03] | 0.0055 | 0.001 |
| Insurance |  |  |  |
| Commercial | Reference | Reference | Reference |
| Medicaid | 1.03 [1.02, 1.04] | 0.0041 | <0.001 |
| Medicare | 1.04 [1.03, 1.05] | 0.0057 | <0.001 |
| Other | 1.12 [1.09, 1.16] | 0.0177 | <0.001 |
| Uninsured | 0.98 [0.97, 0.99] | 0.0049 | <0.001 |
| Elixhauser comorbidities |  |  |  |
| 0 | Reference | Reference | Reference |
| 1 | 1.23 [1.22, 1.24] | 0.0052 | <0.001 |
| 2 | 1.40 [1.39, 1.41] | 0.0061 | <0.001 |
| 3 | 1.53 [1.52, 1.55] | 0.0075 | <0.001 |
| ≥4 | 1.74 [1.72, 1.75] | 0.0090 | <0.001 |
