# Supplementary material for: Primary care utilization among telehealth users and non-users at a large urban public healthcare system
Source: PLoS One. 2022 Aug 5;17(8):e0272605. doi: 10.1371/journal.pone.0272605 (PMC9355262; doi:10.1371/journal.pone.0272605)
Supplement: Attachment — Submitted filename: Response to Reviewers.docx [file pone.0272605.s002.docx]

Dear Dr. Esther Cubo Delgado and Reviewer #1,

Thank you for your comments and the opportunity to revise our manuscript, “Primary care utilization among telehealth users and non-users at a large urban public healthcare system”. We have incorporated your feedback into our manuscript. We feel these changes have improved the quality of the manuscript, particularly in making the discussion and contextualization of findings in existing literature more robust. Below is a point-by-point response for each of your comments. We hope you also find these changes have substantially improved our submission.

Thank you, and please let me know if you have any questions!

Sincerely,

Kevin Chen, MD, MHS (Corresponding Author)
Senior Director, Design & Evaluation
New York City Health + Hospitals
50 Water St, Room 1659
New York, New York 10004
Email: [research@kchenmd.com](mailto:research@kchenmd.com)
Telephone: 929-266-8711

---

**Additional Editor Comments:** In this article, the authors retrospectively compared the number of telehealth visits vs. in-office visits in a large sample in New York.

1. **Comment:**
   For international readers, it would be very convenient to define the different types of health care provider systems used in the US and included in this article.

   **Response:**
   As suggested, we have defined what we are referring to when discussing telehealth. Please see our response to Reviewer #1, Comment #1 for full details.

   **Change:**
   Please see our response to Reviewer #1, Comment #1 for full details.
2. **Comment:**
   As an academic editor I completely agree with the comments provided by the first reviewer. I would discuss the limitations more deeply because it would give an idea how to extrapolate the results to other communities.

   **Response:**
   We appreciate your and the reviewer’s comments on expanding the Limitations discussion. We have added to the Limitations section in response to Reviewer #1, Comments #4-6.

   **Change:**
   Please see our response to Reviewer #1, Comments #4-6 for full details.
3. **Comment:**
   It is very important to understand how the sample was selected (inclusion and exclusion criteria), and to add other confounding variables if they are known (distance to the health care facility).

   **Response:**
   We have clarified what the inclusion and exclusion criteria were in response to Reviewer #1, Comment #2. Unfortunately, additional confounding variables as suggested in Reviewer #1, Comment #4 were not available, but we did include further discussion of this in the Limitations.

   **Change:**
   Please see our response to Reviewer #1, Comments #2 and #4 for full details.
4. **Comment:**
   Other important aspect for publishing a research article is the discussion, comparing the authors´ results with the previous literature. Surprisingly, there are very few references.
   I would suggest the authors to include all the comments provided in this feed back and to send the article again for a second review.

   **Response:**
   Thank you for this suggestion. We have added more comparisons to existing literature and expanded our discussion with more information about our hypotheses on interpretations of our findings. We have also added more citations throughout the text. **Change:**
   In Discussion, added text underlined, changed text stricken:
   ~~This~~ Our findings contrast~~s~~ with ~~findings~~ those of prior studies that included primary care ~~of~~ during a contemporary period which showed no increase in utilization [~~3~~6-~~5~~8], though our study differs in methodology and population demographics.

We uniquely focus on utilization in primary care only whereas prior literature that included primary care [6-8] aggregated outpatient specialties together. The impact of telehealth on different service lines may be different; for example, compared with historical averages, behavioral health services may have experienced rapid adoption and increased total visit volumes with telehealth during the pandemic [6], whereas surgical specialties may have had slower adoption and depressed visit volumes despite telehealth availability during the pandemic [9]. We also chose not to use a historical comparison for visit volume since total visit volumes during the study period were affected by the pandemic. Another difference is in patient demographics; for example, in prior studies that included primary care, 30-77% of patients were White, 8-40% Hispanic, 6-8% Black, and 4-10% Asian [6-8]. In contrast, 9% of our patients were White, 34% Black, 34% Hispanic, and 7% Asian. One study of a large, integrated healthcare system demonstrated that later in the pandemic, while total visit volumes generally returned towards historical trends, not all age, racial, or income groups had similar trajectories [7].

[…] Additionally, since primary care is often the first point of contact for ambulatory healthcare, this increased access may have a specialty-specific effect of increasing utilization.

In references:
1.	Reed ME, Huang J, Parikh R, Millman A, Ballard DW, Barr I, et al. Patient-Provider Video Telemedicine Integrated With Clinical Care: Patient Experiences. Ann Intern Med. 2019 Aug 6;171(3):222-224.

2.	Reed M, Huang J, Graetz I, Muelly E, Millman A, Lee C. Treatment and Follow-up Care Associated With Patient-Scheduled Primary Care Telemedicine and In-Person Visits in a Large Integrated Health System. JAMA Netw Open. 2021 Nov 1;4(11):e2132793.
5.	Quan H, Sundararajan V, Halfon P, Fong A, Burnand B, Luthi JC, et al. Coding algorithms for defining comorbidities in ICD-9-CM and ICD-10 administrative data. Med Care. 2005 Nov;43(11):1130-9.
9. Chao GF, Li KY, Zhu Z, McCullough J, Thompson M, Claflin J, et al. Use of Telehealth by Surgical Specialties During the COVID-19 Pandemic. JAMA Surg. 2021 Jul 1;156(7):620-626.
11.	Fortuna D, Berti E, Moro ML. Multimorbidity epidemiology and health care utilization through combined healthcare administrative databases. Epidemiol Prev. 2021 Jan-Apr;45(1-2):62-71.

12.	Reed ME, Huang J, Graetz I, Lee C, Muelly E, Kennedy C, et al. Patient Characteristics Associated With Choosing a Telemedicine Visit vs Office Visit With the Same Primary Care Clinicians. JAMA Netw Open. 2020 Jun 1;3(6):e205873.

---

**Reviewer #1:** This paper discussed about the association between the use of telehealth and health care utilization. This topic is important especially during crisis (e.g. COVID). The paper is also very concise. My comments are as follows:

1. **Comment:**
   To benefit international readers, the authors may want to describe more how telehealth is used in these clinics. Telemedicine ranged from simply transmission of observations (e.g. blood pressure readings) to full consultations. When the authors said telehealth users, what does this include?
   Will primary care clinic visits be advised after a telehealth consultation? That will explain the difference of no of clinic visits between the both groups. (e.g. skin lesions that cannot be seen clearly during the telehealth consultation)
   Similarly, will telehealth be more likely advised (by healthcare professionals) when the diseases are poorly controlled? that will explain the difference between the two groups

   **Response:**
   Thank you for this comment; we have added a more specific definition in the introduction of what services we are referring to under “telehealth services”. Briefly, we are referring to synchronous audio-only and audio-video care episodes between patients and clinicians for the purpose of medical evaluation and management of acute, chronic, or preventive care needs. To make it clearer that we are discussing such encounters, we have also exchanged the word “services” for “visits” (i.e. telehealth visits instead of telehealth services) to emphasize the analogy to traditionally in-person primary care visits.

   Regarding the reviewer’s question of whether clinic visits will be advised after a telehealth visit, that is one of the key questions we are trying to explore. Thus, we have added a sentence in the introduction addressing this. We caveat that more visits are not necessarily bad—it may be that previously, patients were not seeking care often enough due to general barriers to care.

   Finally, we have added a few references to expand on the theoretical benefit of telehealth visits and a similar use case to ours.

   **Change:**
   In Introduction, paragraph 1, added text underlined, changed text stricken:
   Telehealth ~~services~~visits (specifically synchronous audio-only and audio-video episodes of care between patients and clinicians) may improve access to care by removing certain barriers such as time and cost related to transportation or logistics for childcare or work coverage [1]. In a primary care setting, these visits can be used to deliver medical evaluation and management for acute, chronic, and preventive care needs—analogous to traditional scheduled in-person visits for initial or follow-up evaluation [2].

   …at the same rate as in-person ~~services~~visits in part due to…

   In references:
   1. Reed ME, Huang J, Parikh R, Millman A, Ballard DW, Barr I, et al. Patient-Provider Video Telemedicine Integrated With Clinical Care: Patient Experiences. Ann Intern Med. 2019 Aug 6;171(3):222-224.

2. Reed M, Huang J, Graetz I, Muelly E, Millman A, Lee C. Treatment and Follow-up Care Associated With Patient-Scheduled Primary Care Telemedicine and In-Person Visits in a Large Integrated Health System. JAMA Netw Open. 2021 Nov 1;4(11):e2132793.

2. **Comment:**
   Any inclusion and exclusion criteria of participants? If the "non-users" include simple patients who have good past health and only consult the clinic for episodic problems (e.g. common cold), of course they will have very few clinic visits.

   **Response:**
   We have expanded our Methods section to more explicitly address inclusion and exclusion criteria. All adult primary care patients were eligible for participation in telehealth, and we included all adult primary care patients who had scheduled visits in the study interval in our analysis. There were no set exclusion criteria for telehealth participation. Clinicians and patients used their discretion when scheduling appointments to determine whether a telehealth visit was appropriate or preferable for a given concern.

   The issue of whether non-users are generally healthier patients with fewer healthcare needs (for example, coming into clinic only for an in-person “annual physical”) is a notable one. We attempted to account for this by stratifying by comorbidity count, a metric that we acknowledge is flawed for defining multimorbidity, but in the absence of disease severity data, we used this as a rough proxy. The number of comorbidities someone has in their medical record may estimate both expected healthcare utilization (i.e. more comorbidities may mean more medical service needs) and historical utilization (i.e. someone who has more engagement with the health system may accrue more diagnosis codes in their chart). We have added text and a citation in the Limitations to discuss this.
    **Change:**
   In Methods, paragraph 1, added text underlined:
   All adult primary care patients were eligible for participation in telehealth, and we included all adult primary care patients who had scheduled visits in the study interval in our analysis. There were no set exclusion criteria for telehealth participation. Clinicians and patients used their discretion when scheduling primary care appointments to determine whether a telehealth visit was appropriate or preferable for a given concern.

   In Limitations:
   We used comorbidity count as a rough proxy for both expected healthcare utilization (more chronic diseases may correlate with more medical service use [11]) and past health system engagement (more encounters with the healthcare system may correlate with more diagnosis codes entered into a patient’s chart) and stratified by comorbidity count to account for these factors.

   In references:
   11.	Fortuna D, Berti E, Moro ML. Multimorbidity epidemiology and health care utilization through combined healthcare administrative databases. Epidemiol Prev. 2021 Jan-Apr;45(1-2):62-71.
3. **Comment:**
   In the tables, I suggest to replace "something else" by "others"

   **Response:**
   We have changed “Something Else” in Table 1 to “Other” as suggested.

   **Change:**

   In Table 1, column 1, added text underlined, changed text stricken:

| Race/Ethnicity |  |  | <0.001 |
| --- | --- | --- | --- |
| White | 11,645 (8.7) | 8,204 (9.0) |  |
| Black | 43,134 (32.2) | 33,621 (36.8) |  |
| Hispanic | 46,877 (35.0) | 31,034 (34.0) |  |
| Asian | 11,366 (8.5) | 4,736 (5.2) |  |
| ~~Something Else~~Other | 20,808 (15.6) | 13,722 (15.0) |  |
| Primary Language |  |  | <0.001 |
| English | 75,231 (56.2) | 52,744 (57.8) |  |
| Spanish | 47,986 (35.9) | 31,883 (34.9) |  |
| ~~Something Else~~Other | 10,613 (7.9) | 6,690 (7.3) |  |

4. **Comment:**
   I think the main weakness is that there are many unmeasured parameters to explain the results. The authors may want to discuss possible important parameters and address/discuss this in more details in the discussion; e.g.
   - disease severity: people with poorly controlled diseases may want more contact time with the healthcare system. Therefore, they may have more telehealth and also more consultations. No of co-morbidities is not exactly the same as disease severity
   - Education level
   - level of social support

   **Response:**
   Thank you for this comment. We have added additional sentences in the Limitations regarding unmeasured confounders such as disease severity, education level, socioeconomic status, and distance to a clinic. Unfortunately, these data were not available for this study. **Change:**
   In Limitations, added text underlined:
   However, telehealth users and non-users may differ in propensity to seek care for reasons unaccounted for by their number of comorbidities or the demographics measured in this study, such as distance to the clinic, education level, or socioeconomic status [12]. Further, we did not account for disease severity, which may also impact the number of encounters.

   In references:
   12. Reed ME, Huang J, Graetz I, Lee C, Muelly E, Kennedy C, et al. Patient Characteristics Associated With Choosing a Telemedicine Visit vs Office Visit With the Same Primary Care Clinicians. JAMA Netw Open. 2020 Jun 1;3(6):e205873.
5. **Comment:**
   As this is a cross-sectional study, the causal relationship cannot be established. it is possible that those patients, who had frequent clinical visits, changed some of the visits to telehealth during COVID pandemic

   **Response:**
   We have added an acknowledgement of the limitations of an observational study design in inferring causality in the Limitations section. We also added a sentence expanding on the point regarding high utilizers switching what would have been in-person visits to telehealth visits in the context of a pandemic. In our response to Comment #4, we addressed the issue of unmeasured factors that may drive that high utilization. Finally, we replaced language that may have implied causality with more associative language.

   **Change:**
   In Abstract, added text underlined, changed text stricken:
   We assessed whether ~~access to~~usage of telehealth ~~affected~~ was associated with differential primary care utilization at a large, urban public healthcare system.

   In Discussion:
   This suggests that availability of telehealth visits in primary care may ~~increase~~ be associated with increased individual primary care utilization.

   In Limitations:
   Our study examines utilization behavior during a pandemic, which may not be applicable to non-pandemic times. It is possible that some patients who were higher utilizers of the health system elected to change some visits to telehealth due to concern about travelling during a pandemic. … We used an observational study design, which limits our ability to infer causality.

   In Conclusions:
   Availability of telehealth services may ~~increase~~ be associated with increased primary care utilization in safety-net clinics.
6. **Comment:**
   The authors may also want to elaborate more about clinical/research implications of their results. RCTs and cohorts maybe a better choice to answer whether implementation of telehealth systems would increase healthcare utilization

   **Response:**
   We have added text to elaborate on the implications of our findings and areas for future research. We agree that RCTs and cohorts may be more robust ways to study associations between telehealth availability and healthcare utilization and mention this as well.  **Change:**
   In Discussion, added text underlined:
   While payors and policymakers may be concerned that telehealth availability may increase healthcare costs via additive service utilization, increased primary care utilization in the context of telehealth availability may not be a negative consequence if patients can have more health concerns addressed in a safe and effective manner. Changes in reimbursement structure in the United States towards more value- or outcomes-based payment may allow health systems and other entities to provide telehealth services while tempering costs for payors regardless of visit count.

Future studies of telehealth and healthcare utilization may consider using prospective or cohort designs to examine associations more robustly. And, further research will be needed on clinical and experiential outcomes of telehealth encounters to inform longer-term policies around provision and reimbursement of telehealth.
